# Effective connectivity in cortical and subcortical visual fear processing networks during fearful face viewing in body dysmorphic disorder

**DOI:** 10.21203/rs.3.rs-10780500/v1

**Published:** 2026-09-16

**Authors:** Gillian Grennan, Angela Fang, Andrea Stocco, Qasim Bukhari, Rangaprakash Deshpande, Reza Tadayonnejad, Jamie D. Feusner

**Affiliations:** 1 Department of Psychology, University of Washington; Seattle WA; 2 McGovern Institute for Brain Research and Department of Brain and Cognitive Sciences, Massachusetts Institute of Technology; Cambridge, MA; 3 Massachusetts General Hospital; Cambridge, MA; 4 TMS Clinical and Research Service, Neuromodulation Division, Semel Institute for Neuroscience and Human Behavior at UCLA; Los Angeles California; 5 Department of Psychiatry and Biobehavioral Sciences, David Geffen School of Medicine at UCLA; Los Angeles, CA; 6 Division of the Humanities and Social Sciences, California Institute of Technology; Pasadena, CA University of California at Los Angeles; Los Angeles CA; 7 Department of Psychiatry, University of Toronto; Toronto, Canada; 8 Centre for Addiction and Mental Health; Toronto, Canada; 9 Department of Women and Children’s Health, Karolinska Hospital, Karolinska Institutet; Stockholm, Sweden

**Keywords:** Body Dysmorphic Disorder, Dynamic Causal Modeling, Neuroimaging, Effective Connectivity, Psychopathology, Fear Processing

## Abstract

Individuals with body dysmorphic disorder (BDD) demonstrate emotional processing biases; however, it is unclear whether differences are related to visual fear procesing network dysfunction. Understanding neural connectivity patterns during visual processing of threatening stimuli could provide insights into mechanisms that maintain BDD symptoms. Participants with BDD (n=32) and healthy controls (n=37) completed a fearful face processing task during fMRI. Using dynamic causal modeling, we compared effective connectivity between groups in connections among regions implicated in fearful face processing: the bilateral pulvinar, amygdala, fusiform face area and the medial orbitofrontal cortex (mOFC). We found through Bayesian model selection that both BDD and control groups were fit by the same connectivity model, with driving input of fearful faces to the pulvinar and amygdala and bidirectional connections between each region. Using parametric empirical bayes to test for group differences, we found strong evidence for higher connectivity from the left amygdala to the left mOFC and within the left FFA as well as reduced connectivity from the right amygdala to the right pulvinar in individuals with BDD. However, we did not observe strong evidence for associations with body dysmorphic symptoms. BDD may be characterized by aberrations in the fearful face viewing network. Future studies could address whether these effects are specific to BDD or may also characterize other disorders with emotional face processing dysfunction.

## Introduction

Body dysmorphic disorder (BDD) is a relatively common and debilitating psychiatric disorder characterized by an obsessive focus on misperceived appearance defects that are generally not noticeable to others (1,2). BDD affects approximately 2% of the general population but is understudied compared to disorders of similar prevalence (3–5).

Individuals with BDD show deficits in emotional processing (6–9). Specifically, individuals with BDD misinterpret others’ neutral-expression faces as angry, and angry faces as disgusted (7), and show attentional biases to faces with disgust expressions (10) compared with healthy controls. In addition, those with BDD are less accurate in recognizing angry, sad and neutral expressions, and slower in recognizing angry, happy, and neutral expressions than healthy controls (11).

Few studies have examined emotional processing dysfunction in BDD using fMRI, but both studies to date have found aberrations in the amygdala and prefrontal cortex (12,13). The amygdala is a central hub for detecting threat and processing emotionally salient stimuli, with higher responses to fearful stimuli (14,15). Rangaprakash and colleagues found that during repeated exposure to fearful faces, healthy controls displayed increasingly enhanced connectivity from the amygdala to the medial prefrontal cortex (mPFC), typical of a temporal limbic response to fearful stimuli (16). Notably, this temporal pattern was absent in participants with BDD, implying impaired feedforward frontolimbic connectivity during fear processing (13). In contrast, another study reported increased amygdala-PFC connectivity and greater left amygdala activity in BDD during negative face viewing (12). Other work has shown right amygdala hyperactivity in response to spatially filtered faces (15), while a similar task found right amygdala hypoconnectivity, reinforcing the amygdala’s role in face-processing dysfunction in BDD (16). Further, extensive research on anxiety disorders in general have shown heightened amygdala responses and altered amygdala-PFC coupling during fearful face processing (17,18). Similarly, increased amygdala reactivity to negative stimuli and reduced top-down regulatory influence from the PFC has been shown in depressed individuals (19,20).

More broadly, anxiety has been linked to altered visual processing (21,22) with evidence suggesting that disrupted amygdala-visual cortex connectivity may lead to hypervigilance and heightened responses to emotional stimuli (19,20). Similarly, connectivity between subcortical stimulus processing regions responsible for rapid, coarse visual threat detection such as the pulvinar (23–25) have been shown to have altered connectivity with the PFC in depressed individuals (26). It is yet untested whether emotional processing deficits evident in BDD are mechanistically influenced by interactions between the limbic system and early visual processing regions.

In addition, individuals with BDD show abnormalities in visual processing, particularly a bias toward high-detail over holistic processing. Neuroimaging studies have found increased activity in temporal and ventral visual stream (VVS) regions compared to healthy controls (12,27,28). Notably, greater VVS activity during own-face viewing has been linked to higher anxiety symptoms in BDD (29). Functional specificity of the fusiform face area (FFA), a key VVS region involved in face processing, has been shown to predicted by its structural connectivity to limbic regions such as the amygdala in both humans (30) and non-human primates (31). The FFA has shown increased connectivity with the amygdala (12) and higher-order regions like the precuneus and posterior cingulate cortex (32) in BDD. It remains unclear whether FFA differences in BDD stem from heightened feedforward input from VVS regions or from intrinsic FFA dysfunction. Additionally, pulvinar hyperconnectivity with higher-order visual areas has been observed in social anxiety disorder (33), suggesting that this bottom-up hyperactivity drives overstimulation in higher-order visual or emotional regulation areas, potentially contributing to altered emotional stimulus processing.

Emotional processing dysfunction in BDD may also involve abnormalities in emotion regulation regions such as the orbitofrontal cortex (OFC). The OFC receives input on emotional salience from the amygdala and VVS, and is associated with emotion regulation involved in stimulus processing by way of feedback connections to the amygdala and pulvinar (34). Connectivity between the amygdala and OFC has been confirmed through structural imaging studies showing both feedforward and feedback connections are conserved across both humans and non-human primates (35–37). The OFC has been implicated in related disorders such as obsessive-compulsive disorder (OCD) (38–42), with OFC hyperactivity (43,44) and amygdala-OFC altered effectivity connectivity (45–47) proposed as mechanisms underlying compulsive behaviors. Additionally, OFC and amygdala volume reductions have been shown in individuals with OCD compared to controls (48). Despite the overlap in compulsive symptoms between OCD and BDD, research on the OFC’s role in BDD remains limited. Existing studies have linked OFC self-connectivity to BDD symptom severity (39), and found left OFC hyperactivity during own- versus other-face viewing in BDD (49). However, the specific role of OFC-limbic connectivity in emotion regulation during face processing in BDD is still unknown.

Understanding the influence of fear and anxiety on connectivity patterns during visual processing may provide insights into neural mechanisms that maintain BDD symptoms. Toward this end, we applied dynamic causal modeling in both BDD participants and healthy controls to investigate differences in effective connectivity between regions involved in fearful face processing, including the pulvinar, amygdala, FFA, and an emotion regulation area, the mOFC (50–52). Our hypotheses regarding individual connections were as follows:

1. Based on prior evidence, we expected that individuals with BDD would show higher arousal to fearful faces compared to healthy controls (6–8). We hypothesized that heightened arousal would manifest in increased feedforward connectivity from the pulvinar to the amygdala, from the amygdala to the FFA, and bidirectional connectivity between the pulvinar and FFA. This is supported by prior evidence in both humans and animals showing that the pulvinar has a key role in amplifying evoked responses in higher order regions and that this coupling between regions will strengthen in response to the emotional valence of the stimuli (34,53); there has also been evidence of pulvinar overfeeding in social anxiety disorder (33), which is closely related to BDD.
2. We hypothesized that individuals with BDD would display increased feedback connectivity from the FFA to the amygdala compared to controls, due to prior evidence showing greater reliance of the VVS, which includes the FFA, in BDD (12,27–29), as well as observed hyperconnectivity of the FFA and amygdala during negative face viewing (12).
3. We hypothesized hyperconnectivity from the amygdala to the mOFC based on prior evidence showing hyperactivity (49) of the OFC in BDD populations in tandem with evidence from OCD studies showing hyperconnectivity of the amygdala and prefrontal cortex (38,39).
4. We hypothesized hypoconnectivity from the mOFC to the amygdala due to diminished inhibitory function of the mOFC on the amygdala in the BDD group based on prior evidence showing dysfunctional emotion regulation in BDD (7,54,55) and differential feedback connectivity between the mOFC and amygdala in social anxiety disorder (56,57).
5. We further postulated that all differences in connectivities involving the amygdala would be lateralized to the right side, as the right amygdala has been shown to mediate fear processing (58).
6. Finally, we hypothesized that observed aberrant effective connectivity estimates in the BDD group would show strong evidence for association with BDD symptom severity.

## Methods and Materials

### Participants

We recruited healthy controls (n = 37) and participants with BDD (n = 32); both groups were comprised of unmedicated, young individuals (aged 15-38 years). The study was approved by the UCLA Institutional Review Board and all research was performed in accordance with relevant guidelines and regulations. Informed consent from all participants ≥18 years old was obtained prior to study procedures, and parental consent plus participant assent was obtained for those under 18. Participants were right-handed as determined by the Edinburgh Handedness Inventory (59) and were deemed to have normal, or corrected-to-normal, visual acuity as tested with a Snellen eye chart. Participants were recruited from the community as well as through advertisements in outpatient clinics at UCLA and in the greater Los Angeles area.

Participants with BDD were excluded if they met diagnostic criteria for any lifetime psychotic or bipolar disorder, current substance dependence/abuse, and/or if they had active suicidal or homicidal ideation or self-injurious behavior. Participants were similarly excluded if they had a lifetime neurological disorder, current pregnancy, any medical illness that could affect cerebral metabolism, or current treatment with cognitive behavior therapy (CBT). Participants were required to be free from psychoactive medications for at least 8 weeks prior to entering the study. One participant with BDD was undergoing outpatient psychotherapy (although not CBT) at the time of the study.

To enhance generalizability, we allowed current DSM-IV diagnoses of dysthymia, OCD, major depression, panic disorder, generalized anxiety disorder, social anxiety disorder, or agoraphobia. Comorbid diagnoses in the BDD group can be found in Supplementary Table 1. Controls were excluded if they had any lifetime DSM-IV Axis I disorder.

### Measures

#### Clinical Measures.

Participants were telephone screened, followed by a clinical interview with the study physician (JDF). Diagnostic assessments included the Mini International Psychiatric Interview (MINI) (60) and the BDD Diagnostic Module (61) based on the DSM-IV. Anxiety and depression symptom severity were measured using the Hamilton Anxiety Rating Scale (HAMA) (62), and the Montgomery Asberg Depression Rating Scale (MADRS) (63). Delusionality was assessed using the Brown Assessment of Beliefs Scale (BABS) (64). Participants with BDD were additionally administered the Yale-Brown Obsessive Compulsive Scale Modified for BDD (BDD-YBOCS) to assess BDD symptom severity (65).

#### Imaging data acquisition.

Functional MRI data were acquired on a Siemens Trio 3T scanner using a T2*weighted echo planar imaging (EPI) gradient-echo pulse sequence (repetition time/echo time = 2,500/25ms; flip angle = 80°; matrix = 64 x 64; field-of-view = 192 mm; in-plane voxel-size = 3mm^3^; 0.75 mm intervening spaces; 32 total interleaved slices). Higher resolution-matched bandwidth (MBW) T2 and T1-weighted images were collected for coregistration. fMRI data preprocessing methods can be found in the Supplementary Materials.

#### fMRI data preprocessing.

fMRI data were preprocessed in the FMRIB Software Library (FSL version 5.0.6). Specifically, data underwent spatial smoothing (5mm full-width-half-maximum Gaussian kernel), high-pass temporal filtering (100s), skull stripping, and were motion corrected using 6 motion parameters. We coregistered participant’s functional images to their MBW T2-weighted structural image, which was then registered to the MNI-152 template brain volume. We excluded participants with motion values >2 standard deviations from the group mean as defined by FSL DVARS (66).

#### Fearful face task.

**Figure 1.**
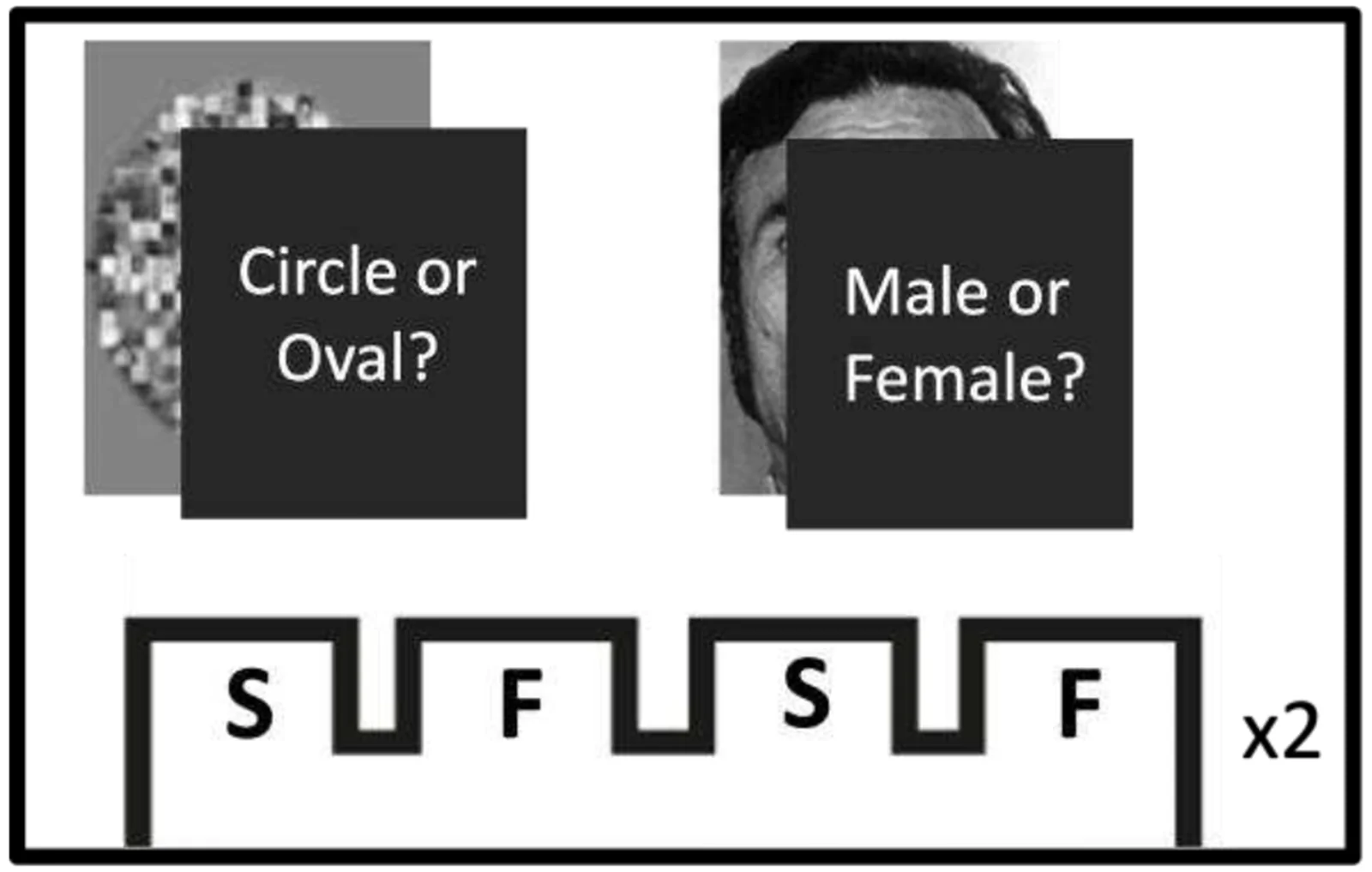
Fearful face processing paradigm. During this task, an equal number of male- and female-appearing faces were presented as either scrambled images or unaltered images of fearful faces. Participants were asked to identify the gender of the face to ensure attention.

Participants viewed blocks of either fearful faces or scrambled shapes while performing a gender-labeling task to ensure vigilance (67) (Figure 1). Face-stimuli were selected from the NimStim dataset which has standardized face images (68). Each block contained 4 stimuli, either faces or scrambled shapes, with equal numbers of male and female faces. Blocks were presented 10 times per condition across 2 runs, totaling 40 trials per stimulus type. Stimuli were shown for 4 seconds each with a 0.5-second interstimulus interval. During face trials, participants identified gender (male/female); during scrambled trials, they identified shape (circle/oval) by pressing corresponding keys.

#### Region of Interest (ROI) Parcellation.

We chose regions of interest (ROIs) based on prior structural and functional evidence of these ROIs as central nodes in a visual fearful face processing network as well as because of their implications in BDD (69). Selected ROIs were as follows: bilateral pulvinar, amygdala, fusiform face area (FFA), and medial orbitofrontal cortex (mOFC). The mOFC was included based on previous findings showing abnormal mPFC-amygdala connectivity in BDD patients (13) as well as OFC differences in BDD during own-face processing (49) and studies implicating the OFC as part of an extended face processing network (50,70). Specifically, the medial, rather than lateral, portion of the OFC was selected due to a greater number of published papers supporting a link between mOFC and fear and human evidence for structural white matter connectivity between the amygdala and mOFC (36,71). Bilateral pulvinar was defined anatomically using the Craddock-200 atlas (72). Bilateral amygdala and mOFC were anatomically defined using Automatic Anatomical Labeling (73). Bilateral FFA was extracted using a functionally-defined Neurosynth reference inference Z map (thresholded at p<0.05) (74). We further validated our extracted FFA ROIs with prior work accounting for inter-individual variability in the functionally-defined FFA and ensured the average size of the ROIs was close to prior, more conservative analyses (75). ROIs were selected based on strong a priori anatomical and functional evidence regarding fearful face processing; given the well-established roles of the ROIs selected, we elected to use anatomical derivations of the ROIs rather than mass-univariate activation maps. This is consistent with recommendations in the DCM literature emphasizing biologically motivated model specification (76). ROI locations were defined individually to account for anatomical variability. Bilateral ROIs were kept separate due to known hemispheric differences in BDD and fear processing (12,13,77,78). Although hypotheses involving the amygdala were lateralized to the right side, we opted to include bilateral nodes to ensure that the model captured the full architecture of fearful face processing. As DCM inferences depend on network completeness, omitting left-side connections could bias the estimation of right-sided parameters or obscure meaningful group differences (76,79). Eigenvariate time series from these regions were then extracted using principal component analysis (PCA) to provide a matrix of size *timepoints x trial type* for each ROI and subject individually; each ROI eigenvariate explained at least 80% of the variance across all participants. ROI timeseries were mean-centered through the eigenvariate extraction process prior to use in the DCM analysis. As an exploratory analysis, we examined univariate task effects using GLM across all ROIs used within the DCM analysis; across all subjects, participants showed greater BOLD fMRI activation across all ROIs in response to fearful versus scrambled stimuli as analyzed via one-sample t-tests (all uncorrected-p < 0.04).

#### Dynamic causal modeling.

**Figure 2.**
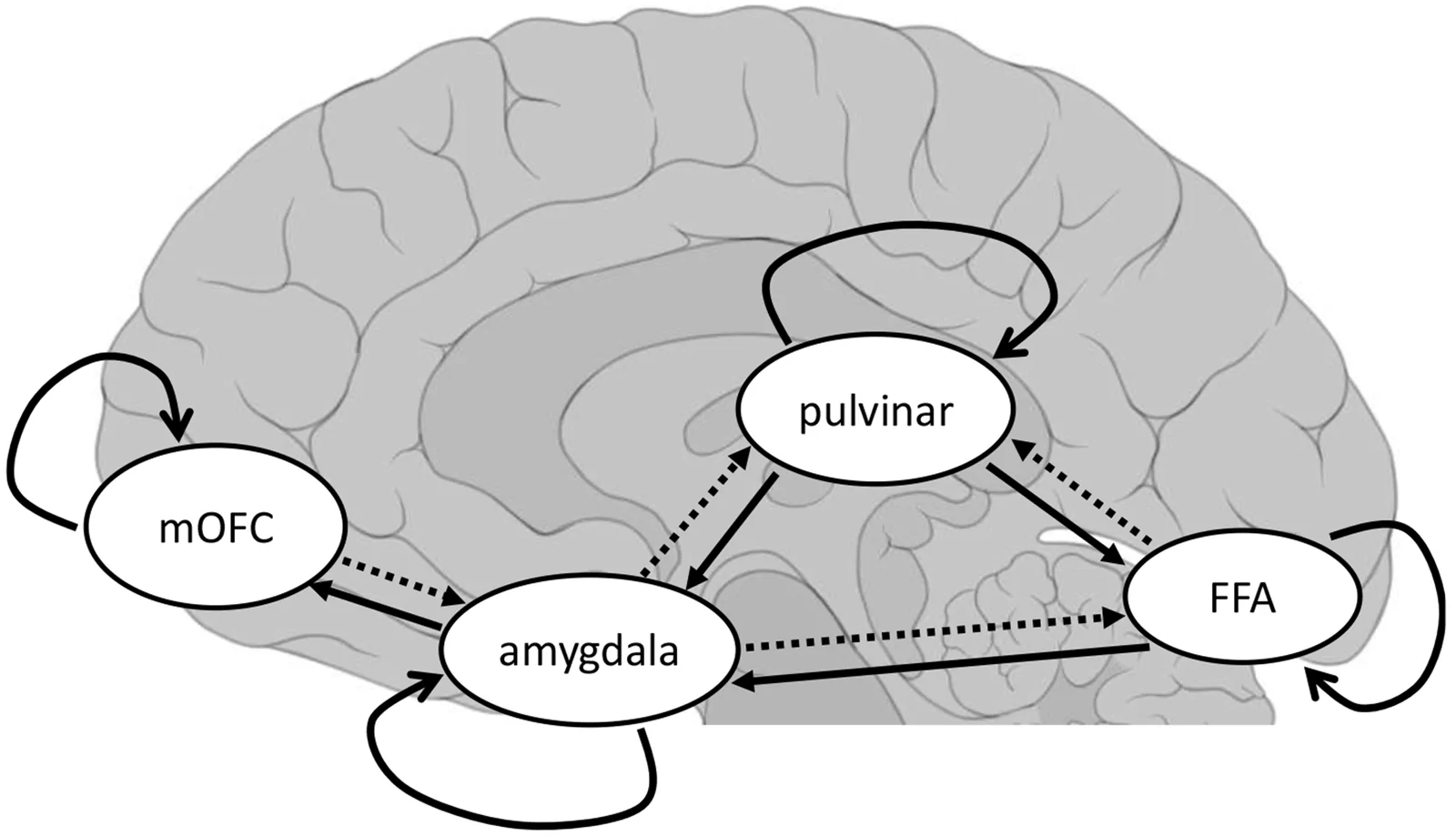
The *a priori* DCM with nodes of the bilateral pulvinar, amygdala, FFA and mOFC. We included connections between the pulvinar, FFA, and amygdala as well as connections between the amygdala and mOFC. We also included intra-region self-feeding connections. Feedforward and intra-region connections between regions are displayed as solid lines while feedback connections are displayed as dashed.

We used dynamic causal modeling (73; DCM) in SPM12 to assess effective connectivity during fearful face processing. Bilinear, non-stochastic DCMs were estimated separately for each subject using eight Regions-of-Interest (ROIs): the left and right pulvinar, amygdala, FFA, and mOFC. An *a priori* model specified the pulvinar as the starting node, which shares connections to both the FFA and amygdala. The FFA similarly connects to the amygdala, which terminally connects to the mOFC (Figure 2). Connections between all nodes were verified to have plausible biophysical basis by examining prior studies showing white matter and functional connectivity between these nodes in humans (34,50,81–84). In total we included 8 feedforward connections, 8 feedback connections, and 8 intra-region connections (Figure 2; 24 connections total). Trial types (scrambled versus fearful) were used as the driving inputs. Importantly, DCM estimates directed connectivity based on a biophysically informed generative model of neural interactions, Thus, the term “directional” reflects the model-based influences of one region on another that are more likely to account for the observed regional timeseries, without directly relying on their observed temporal precedence (which might be affected by regional differences in the hemodynamics response: (76,80). Therefore, our interpretation of DCM results is grounded in this model-based framework, rather than assumptions about temporal causality. Model comparison was conducted using Bayesian random-effects analysis, which accounts for between-subject heterogeneity in the model that provides the best fit to the data. Model comparison was performed within each group separately, to further account for any underlying heterogeneity that would affect model fit. This procedure yields, for each model, the probability densities that a participant from that group will be best fit by a given model. These distributions were then used to compute the exceedance probability associated with each model, that is, the probability that a given model would fit an individual better than any of the others (85). A DCM model includes different matrices of parameters, which capture different forms of direct and modulatory interactions between ROIs or between experimental stimuli and ROIs. The connectivity parameters discussed throughout the manuscript relate to the A-matrix, or the matrix of direct intrinsic connectivity between ROIs as visualized in Figure 2. We opted to focus on the A-matrix (to represent stable group differences in intrinsic connectivity) and the C-matrix (to represent stable regional differences in responses to specific stimuli) to address our hypotheses. We did not include the B-matrix (which reflects modulatory effects from the experimental stimuli) or the D-matrix (which reflects modulatory effects of one region on the connectivity), as these were not central to addressing our hypotheses. Given this, we decided instead to only include the direct effects of stimuli on different ROIs, and systemically explore the best ways to connect the inputs to the model (see Supplementary Figure 1); we have followed similar procedures in prior work (86,87).

#### Statistical analysis.

Group-level analyses were conducted using Parametric Empirical Bayes (PEB) within the DCM framework. PEB provides a hierarchical Bayesian approach for estimating between-subject effects by accounting for the uncertainty of individual (first-level) DCM parameters and modeling group-level effects in a single Bayesian framework (88). This approach is the recommended standard for group comparisons in DCM as it enhances sensitivity, avoids circularity, and eliminates the need for traditional multiple-comparison correction applied to pairwise-tests across individual parameters (89,90). To estimate group differences across parameters we used the estimated A-matrix from each participant’s DCM. The posterior means and covariance estimates of all subject-wise parameters also taken forward for the second-level PEB models. We then used Bayesian Model Reduction (BMR), which efficiently searches the model space by pruning parameters that do not contribute to model evidence. The results models were averaged using Bayesian Model Averaging (BMA) to yield robust posterior estimates of group effects, weighted by their evidence. Connectivities with a posterior probability (*Pp*) > 0.95 are considered as having strong evidence for group differences. The full estimated A-matrix as well as the PEB results for each estimated connectivity parameter are reported in Supplementary Table 1.

To test associations between connectivity estimates and symptom severity, we conducted a PEB analysis within individuals with BDD only, looking at the relationship between with all 24 connectivity parameters and BDD-YBOCS total scores. We also performed further exploratory analyses using dimensional measures of anxiety and depression symptoms.

## Results

### Bayesian model selection.

The model space was defined to address: (A) which region in the visual fearful face processing network is the input region for presented stimuli and if this varies depending on whether stimuli were fearful vs scrambled faces, and (B) the directionality of these connections, i.e., whether this network is operating as a feedback, feedforward, or bidirectional system. We conducted a two-stage model comparison to address these questions. First, we compared models that differed by stimulus driving regions, that is, the input regions for the different stimulus types (Supplementary Figure 1A). We compared a model in which (i) both stimulus types (fearful vs scrambled) drove activity in the pulvinar (Pulvinar/Pulvinar model), (ii) fearful faces drive input to both the pulvinar and amygdala, while scrambled faces only drive activity in the pulvinar (Pulvo-amygdala/Pulvinar model), and (iii) fearful faces drive activity in the amygdala while scrambled faces drive activity in the pulvinar (Pulvinar/Amygdala model). We determined through Bayesian model selection(91) that both groups (BDD and HCs) were best fit by the Pulvo-amygdala/Pulvinar model, in which fearful faces drove regional input activity in the bilateral pulvinar and amygdala while the scrambled faces drove input activity in just the bilateral pulvinar. Using these verified stimulus driving regions, we then tested models with varying directionality of connections using a (i) feedforward model, (ii) feedback model, and (iii) bidirectional model, in which all connections between nodes were reciprocal (Supplementary Figure 1B). Again, using Bayesian model selection, we observed that both groups were fit by the model with bidirectional connectivity between all ROIs, providing evidence for both feedforward and feedback connections utilized within this network. Exceedance probabilities for each model within each group can be seen in Supplementary Figure 1C. The distribution of the exceedance probabilities for each model can be seen in Supplementary Figure 2.

### Group differences in effective connectivity

**Figure 3.**
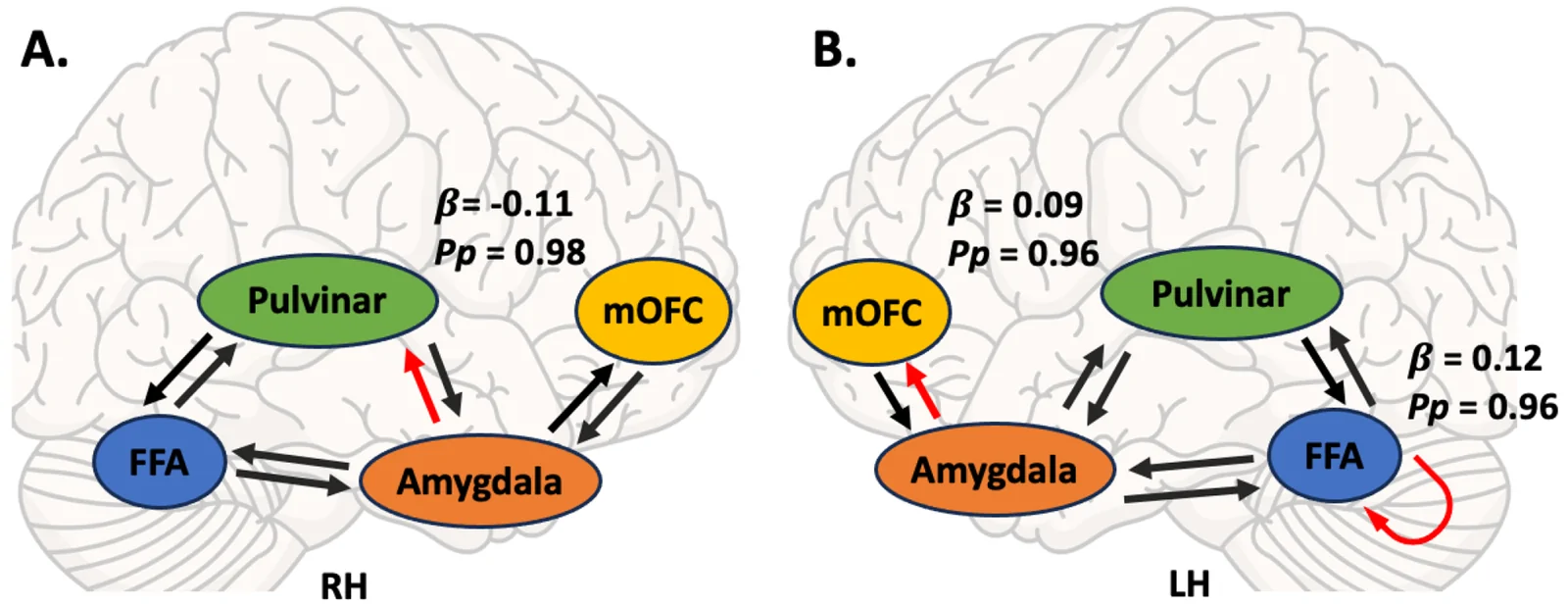
PEB analysis yielded strong evidence for differences in three effective connectivity estimates between individuals with BDD and HCs. A) effective connectivity from the right amygdala to the right pulvinar (*β* = −0.11, *Pp* = 0.98) was reduced in individuals with BDD compared to HCs. B) effectivity connectivity from the left amygdala to the left mOFC (*β* = 0.09, *Pp* = 0.96) as well as connectivity within the left FFA (*β* = 0.12, *Pp* = 0.96) was greater in individuals with BDD compared to controls. RH = right hemisphere; LH = left hemisphere; BDD=body dysmorphic disorder; HC=healthy controls; mOFC=medial orbitofrontal cortex; FFA=fusiform face area

As described above, both groups were fit by the same structural model in which scrambled faces drove input to the pulvinar, fearful faces to the pulvinar and amygdala, with bidirectional connections between all nodes. Modeling only ipsilateral and self-connections, the final model included 24 connections. Using Parametric Empirical Bayes (PEB), we investigated group differences across the estimated connectivity parameters (see Figure 3, Supplementary Table 1). We observed strong evidence for greater connectivity from the left amygdala to the left mOFC (*β* = 0.09, *Pp* = 0.96) and greater connectivity within the left FFA (*β* = 0.12, *Pp* = 0.96), in individuals with BDD compared to controls. Additionally, we observed strong evidence that individuals with BDD showed reduced connectivity from the right amygdala to the right pulvinar (*β* = −0.11, *Pp* = 0.98). No other connectivity parameter yielded differences with strong evidence (all other *Pp* < 0.81).

### Associations with body dysmorphic symptoms

There were no associations with strong evidence of likelihood with body dysmorphic symptoms within the BDD group across all estimated connectivities, however we did observe a moderate association with symptom severity and connectivity within the left pulvinar (*β* = −0.01, *Pp* = 0.94) and from the right pulvinar to the right FFA (*β* = 0.01, *Pp* = 0.94).

### Exploratory associations with depression and anxiety symptoms

Within the BDD group, we observed an association with strong evidence between the connectivity estimate from the left amygdala to the left mOFC and anxiety symptoms (*β* = 0.01, *Pp* = 0.97), such that higher connectivity was associated with increased anxiety symptoms; this connectivity was moderately associated with depression symptoms in the same direction (*β* = 0.01, *Pp* = 0.93). Reduced connectivity within the left pulvinar was shown to have strong evidence for association with increased depression symptoms (*β* = −0.01, *Pp* = 0.97), an effect that was moderate for anxiety symptoms (*β* = −0.01, *Pp* = 0.94). We observed moderate associations with anxiety symptoms and the connectivities from the left FFA to the left amygdala (*β* = −0.01, *Pp* = 0.94), and from the left pulvinar to the left FFA (*β* = 0.01, *Pp* = 0.93). Similarly, we observed moderate associations with depression and the connectivities from the left pulvinar to the left FFA (*β* = 0.01, *Pp* = 0.92) and from the right FFA to the right amygdala (*β* = −0.01, *Pp* = 0.93).

## Discussion

This study examined effective connectivity involved in fearful face processing in individuals with BDD and HCs. We observed strong evidence for higher connectivity from the left amygdala to the left mOFC and within the left FFA as well as reduced connectivity from the right amygdala to the right pulvinar in individuals with BDD compared to healthy controls. While aberrant connectivity from the left amygdala to the left mOFC showed moderate-strong evidence for a positive association with anxiety and depression symptoms in individuals with BDD, we did not observed associations with BDD symptoms with strong evidence of likelihood across all connectivity parameters. Additionally, no single hypothesized connection showed strong evidence for group differences.

Feedforward connectivity from the left amygdala to the left mOFC was strongly evidenced to be different between patients and controls with connectivity heightened in BDD compared with HC. We hypothesized that there would be increased amygdala-to-mOFC connectivity in BDD, lateralized to the right hemisphere, given the right amygdala’s role in fear processing (58), which was not confirmed. However, the left amygdala has been associated more generally with emotional processing (78,92). Further, while the right amygdala is involved in the rapid detection of emotional/ threatening stimuli, the left amygdala is responsible for more detailed stimulus evaluation deriving from stimuli-induced arousal fluctuations (93,94). Thus, we speculate that the current findings could possibly reflect deficits in emotional processing for individuals with BDD, arising during a secondary process of stimulus evaluation.

Several studies have reported distinct amygdala-prefrontal connectivity patterns in BDD compared to HCs, both during task and at rest (12,13,95). In the same participants as the current study, a self-emotion labeling task revealed impaired feedforward amygdala-mPFC connectivity in BDD; unlike HCs, individuals with BDD showed no increase in connectivity across experimental blocks (13). Another study found increased left amygdala-prefrontal connectivity and hyperactivity in the left amygdala during negative face viewing in BDD (12). Similarly, amygdala hyperactivity, particularly on the right, was observed in response to emotionally neutral, spatial frequency-filtered faces (27). At rest, BDD participants also showed greater left amygdala-frontal connectivity compared to HCs (95).

These findings support disrupted frontolimbic connectivity in BDD, especially during emotional face perception (13). Notably, similar frontolimbic hyperconnectivity has been reported in social anxiety disorder (96), a condition that shares features with BDD, such as heightened social rejection sensitivity and self-focused attention (6,7,97–100). Differences in OFC activity (101) and connectivity (39,47,102) has been observed in OCD compared with HC, and linked to emotion regulation during stimulus appraisal as well as compulsive subtypes of OCD. Future work, potentially in a transdiagnostic sample of BDD, social anxiety disorder, and OCD, could potentially help elucidate shared and unique phenotypes and any potential associations between altered frontolimbic connectivity and cross-cutting symptom domains.

Moreover, connectivity from the right amygdala to the right pulvinar showed strong evidence in distinguishing BDD from HC, with reduced connectivity in BDD compared with HC. Although outside of our hypotheses, one speculative possibility is that this might reflect diminished attention modulation in response to threat in individuals with BDD. The pulvinar, a core region of the thalamus, is thought to provide salience-dependent stimulus information to areas such as the amygdala and OFC via feedforward connections. The pulvinar also receives a wide array of feedback input from the cortex and integrates this multimodal information to redirect processing towards salient stimuli (34,53). Diminished amygdala-pulvinar feedback connectivity could possibly be related to symptoms of attentional bias previously seen in individuals with BDD (10,103–105). However, this remains speculative until directly tested since this connection, to the best of our knowledge, has not been previously probed in those with BDD.

Further, we observed strong evidence for higher connectivity within the left FFA in individuals with BDD compared to HCs. Prior research on FFA activation and connectivity differences in BDD are mixed, with some showing higher overall FFA activity (28) and connectivity (12) in BDD compared with HC, and other studies showing no differences in FFA activation level in response to neutral faces in BDD (12,27). Our results converge on growing evidence showing face processing differences, specifically negative emotional face processing, in BDD centered around deficits in the FFA (12,77). Contrary to our hypotheses, we did not observe aberrant FFA connectivity within other regions such as FFA-amygdala or FFA-pulvinar connectivity in the BDD group compared to controls. Hyperactivity and hyperconnectivity of the FFA in BDD has been interpreted as an overreliance on the VVS, or high-detail visual processing, compared to HC (28,77). It is possible that we did not observe FFA connectivity differences with regions such as the amygdala due to the specific task design. The task of gender labeling, although of low task difficulty but requiring facial characteristic identification, could have potentially required both groups to rely on detail processing by highly engaging the VVS (106) and its connections with the pulvinar and amygdala, making inherent connectivity differences in VVS regions such as the FFA too subtle for accurate group distinction (i.e., a ceiling effect).

Notably, Bayesian model selection revealed that both groups were best fit by the same structural connectivity model, with bidirectional connections between regions of the pulvinar, amygdala, FFA, and mOFC. This indicates that individuals with BDD display a comparable combination of feedforward and feedback influence during fearful face processing as is typical in HC (107–109).

Several limitations should be noted. Although showing strong evidence, group differences showed modest effect sizes and aberrant connectivities did not show strong evidence for associations with symptom severity in BDD. However, our sample showed relatively low variance in BDD symptoms, likely due to the small sample size. Null symptom-brain associations are not uncommon in clinical connectivity studies, particularly in relatively small samples with restricted symptom variance (110,111); this emphasizes the need for larger samples to enable us to capture sufficient symptom heterogeneity within our clinical sample. Further, we utilized scrambled images as our control condition rather than neutral faces, given prior evidence that face—baseline contrasts are more reliable (112–116). However, this limits the ability to disentangle the effects of fearful expressions from general face processing. Future studies should include neutral and other emotional expressions as well as incorporate retest reliability to enhance validity. Additionally, the task used in this study was an implicit face processing paradigm, which limits the generalizability of our findings to ethological BDD symptoms. As participants were not explicitly instructed to evaluate the emotional content of the faces, the extent to which the observed effects reflect deliberate emotional appraisal versus automatic perceptual or attentional processes remains unclear. Finally, we constrained our regions and connections of interest for this study to ones we believed to be implicated in BDD and important for fearful face processing, but there could exist relevant regions outside of this network.

The BDD sample included many individuals with comorbidities (n=17/32, see Table 1) to allow for a more generalizable sample, given the high rate of psychiatric comorbidities in the general BDD population (117,118). As some connections showed strong evidence for positive associations with anxiety and depression symptoms in the BDD group, there may still be latent factors related to comorbidities that drove our group differences, which is an inherent limitation in case-control studies. Finally, both groups were comprised of majority female participants. As BDD has been shown to occur at almost equal rates across genders (2), this limits the generalizability of our findings.

This study is part of a growing effort to investigate the neural mechanisms underlying emotion and face processing in individuals with BDD. We found that during fearful face viewing, connectivity between the amygdala and mOFC, within the FFA, and between the amygdala and pulvinar showed strong evidence for differences between individuals with BDD from HC. Of note, none of our hypothesized connections showed strong evidence for group differences. Clinically, this network-level characterization may help guide future interventions aimed at reducing perceptual threat bias or through strengthening regulatory prefrontal feedback. For instance, connectivity-informed targets could be leveraged in neuromodulation augmentation approaches (i.e., TMS, neurofeedback) or used to evaluate mechanisms of change across existing treatments such as cognitive-behavioral therapy. Our findings add to the current theoretical model of BDD, further implicating the amygdala and FFA, and their connections, as regions with key differences compared to neurotypical individuals. Future work should investigate the role these regions play in the etiology or in the maintenance of BDD. Future research is warranted to better understand if and how aberrant fearful face viewing is linked to specific symptoms, including utilizing transdiagnostic samples that could potentially overlap in symptom dimensions and neural phenotypes.

## Supplementary Material

This is a list of supplementary files associated with this preprint. Click to download.

• SRsupplementREVISEDfinal.docx

## Figures and Tables

**Table 1.** Demographic and clinical characteristics of the sample.

Demographics				
Characteristic	BDD group (n = 32)	Control group (n = 37)	Test statistic	P-value
Age	23.50 ± 0.84	21.68 ± 0.74	t = 1.63	0.11
Sex (n, %)			t = −0.24	0.81
Female	27 (84.38)	32 (86.49)		
Male	5 (15.62)	5 (13.51)		
Race/ethnicity (n, %)			*χ*^2^ = 2.10	0.72
Asian	9 (28.12)	9 (24.32)		
African American/Black	2 (6.25)	6 (16.22)		
Hispanic	2 (6.25)	1 (2.70)		
Mixed race	3 (9.38)	3 (8.11)		
White	16 (50)	18 (48.65)		
Education (years)	15.13 ± 0.57	13.85 ± 0.45	t = 1.78	0.08
BDD-YBOCS	29.50 ± 0.97	NA		-
BABS	15.19 ± 0.58	NA		-
MADRS	15.53 ± 1.40	1.22 ± 0.27	t = 10.70	**<0.0001**
HAMA	10.06 ± 1.18	2.16 ± 0.29	t = 6.94	**<0.0001**
Comorbid diagnoses (n, %)				-
Agoraphobia	2 (6.25)			
Dysthymia	2 (6.25)			
Generalized anxiety disorder	4 (12.5)			
Major depressive disorder	10 (31.3)			
Social anxiety disorder	4 (12.5)			
Panic disorder	1 (3.13)			

P-values are derived from between-groups t-tests or chi-square (marked with *). Comorbidities of BDD group are also listed here. To enhance generalizability, we allowed current DSM-IV diagnoses of dysthymia, OCD, major depression, panic disorder, generalized anxiety disorder, social anxiety disorder, or agoraphobia. Of note, 5/32 (15.63%) of participants in the BDD group had at least 2 comorbidities.
